# Supplementary material for: The causal role of circulating inflammatory markers in osteoporosis: a bidirectional Mendelian randomized study
Source: Front Immunol. 2024 Jul 18;15:1412298. doi: 10.3389/fimmu.2024.1412298 (PMC11291241; doi:10.3389/fimmu.2024.1412298)
Supplement: Supplementary file 1 [file DataSheet_1.zip › supplementary material/CIM-OSTEOPOROSIS /GCST90274825.csv_forest.pdf]

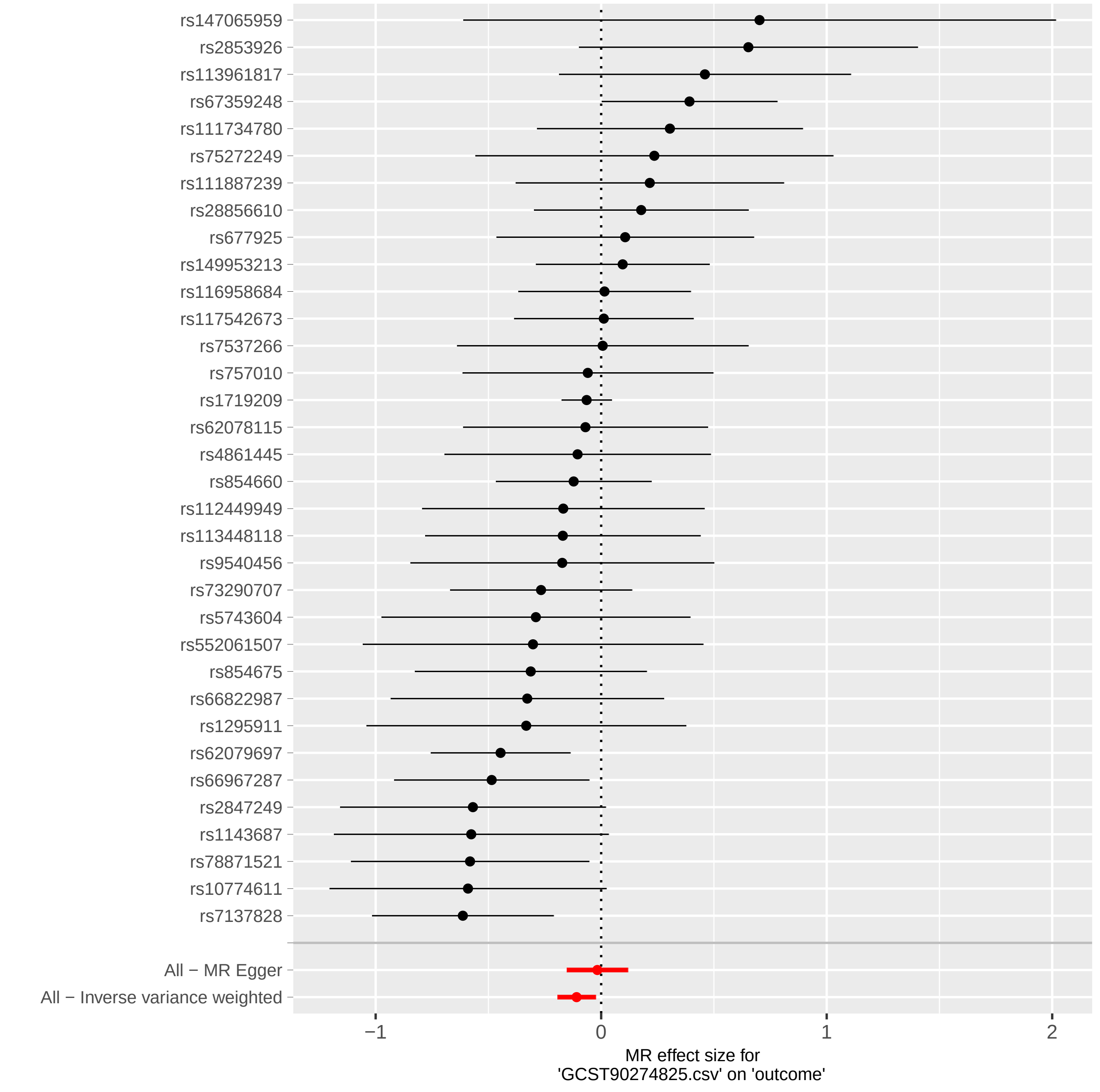
rs147065959
rs2853926
rs113961817
rs67359248
rs111734780
rs75272249
rs111887239
rs28856610
rs677925
rs149953213
rs116958684
rs117542673
rs7537266
rs757010
rs1719209
rs62078115
rs4861445
rs854660
rs112449949
rs113448118
rs9540456
rs73290707
rs5743604
rs552061507
rs854675
rs66822987
rs1295911
rs62079697
rs66967287
rs2847249
rs1143687
rs78871521
rs10774611
rs7137828
All − MR Egger
All − Inverse variance weighted
−1
0
1
2
MR effect size for
'GCST90274825.csv' on 'outcome'
